# Light-Output Enhancement of GaN-Based Light-Emitting Diodes with Three-Dimensional Backside Reflectors Patterned by Microscale Cone Array

**DOI:** 10.1155/2014/837586

**Published:** 2014-07-15

**Authors:** Huamao Huang, Jinyong Hu, Hong Wang

**Affiliations:** Engineering Research Center for Optoelectronics of Guangdong Province, Department of Physics, School of Science, South China University of Technology, Guangzhou, Guangdong 510640, China

## Abstract

Three-dimensional (3D) backside reflector, compared with flat reflectors, can improve the probability of finding the escape cone for reflecting lights and thus enhance the light-extraction efficiency (LEE) for GaN-based light-emitting diode (LED) chips. A triangle-lattice of microscale SiO_2_ cone array followed by a 16-pair Ti_3_O_5_/SiO_2_ distributed Bragg reflector (16-DBR) was proposed to be attached on the backside of sapphire substrate, and the light-output enhancement was demonstrated by numerical simulation and experiments. The LED chips with flat reflectors or 3D reflectors were simulated using Monte Carlo ray tracing method. It is shown that the LEE increases as the reflectivity of backside reflector increases, and the light-output can be significantly improved by 3D reflectors compared to flat counterparts. It can also be observed that the LEE decreases as the refractive index of the cone material increases. The 3D 16-DBR patterned by microscale SiO_2_ cone array benefits large enhancement of LEE. This microscale pattern was prepared by standard photolithography and wet-etching technique. Measurement results show that the 3D 16-DBR can provide 12.1% enhancement of wall-plug efficiency, which is consistent with the simulated value of 11.73% for the enhancement of LEE.

## 1. Introduction

Low light-output efficiency is one of the biggest obstacles for the extensive use of GaN-based light-emitting diodes (LEDs) in general lighting. To enhance the light-output of the top side in LED chips, a backside reflector is used [1–3]. However, the extensively used reflector always consists of multiple thin-films, with which the LED chip makes up planar optical waveguides. Thus, lights outside the critical angle would be confined within the device and be repeatedly reflected by the GaN/air interface. These lights would be absorbed by semiconductor materials, multiple quantum well, and metal electrodes, which would finally convert to heat. Therefore, the light-output efficiency is suppressed. In order to break the planar structure, three-dimensional (3D) reflectors can be used.

The microscale tetragonal-lattice of SiO_2_ pyramid with silver (Ag) mirror was first proposed to be placed on the surface of p-GaN in flip-chip LEDs [4]. In conventional LED chips, the metal mirror was also adopted. After the sapphire substrate was wet-etched in hot acid, an Ag layer was deposited on the backside of the textured sapphire as 3D reflector [5]. However, the metal Ag thin-film is not advisable to be directly attached on sapphire substrate because of the poor adhesion performance [6], while the distributed Bragg reflector (DBR) composed of dielectric multilayer films is preferred. Before the DBR is fabricated, the textured sapphire can also be created by laser interference lithography followed by dry-etching [7]. However, the etching of sapphire substrate is not easy no matter whether the wet-etching [5] or dry-etching [7] was employed. Alternatively, SiO_2_ nanostructures were used to form the pattern, and then the mirror layer was deposited on the 3D surface [8–10]. The SiO_2_ nanospheres were spun-casted on a benzocyclobutene (BCB) layer and the bottom half of the nanospheres were embedded in BCB after annealing; subsequently, nanoscale convex or concave pattern can be created on the condition that the nanospheres remained or were wiped off [8]. The SiO_2_ nanosphere monolayer can also be covered by a thick SiO_2_ layer, and the fluctuated surface is the pattern for 3D reflector [9, 10]. However, the nanosphere lithography is hard to realize wafer-scale SiO_2_ monolayer pattern in mature production line. Thus, the microscale structures defined by traditional photolithography are more suitable for mass production. A microscale pyramid array of DBR embedded in epilayer was studied [11]. However, this approach would affect the epitaxial process.

In this paper, a SiO_2_ thin-film was prepared at the backside of sapphire substrates. Then, the triangle-lattice of microscale SiO_2_ cone array was patterned by standard photolithography and wet-etching technique. After that, a 16-pair Ti_3_O_5_/SiO_2_ DBR was deposited to build 3D reflector. The light-output enhancement is demonstrated by numerical simulation and experiments.

## 2. Materials and Methods

**Figure 1:**
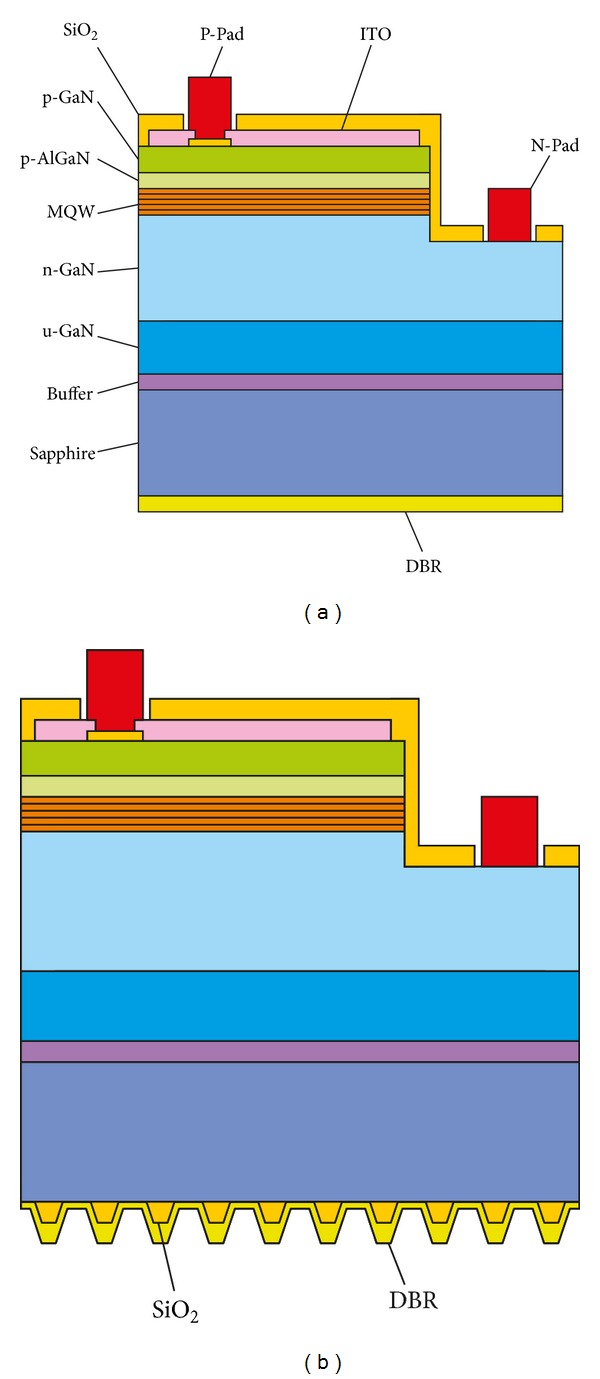
Schematic structure of LED chips with (a) flat DBR and (b) 3D reflector.

Figure 1 shows the LED chip with conventional flat reflector and that with our proposed 3D reflector. For conventional flat reflector in Figure 1(a), a 16-pair DBR layer is directly deposited on the backside of sapphire substrate. For our proposed 3D reflector in Figure 1(b), a triangle-lattice of SiO_2_ cone array is placed on the backside of sapphire substrate at first and followed by the 16-pair DBR later.

### 2.1. Simulation

**Figure 2:**
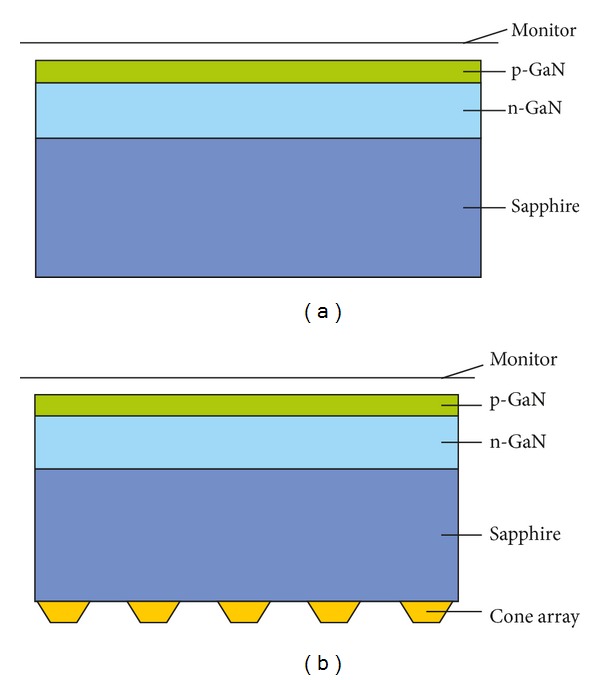
Sketch of side-view of simulation model with (a) flat reflector and (b) 3D reflector.

The side-view of simulation model is sketched in Figure 2. The chip size is 254 *μ*m (width) × 584.2 *μ*m (length) × 106.3 *μ*m (height), in which the height of cone array is not reckoned. In order to reduce the computation resource, this 3D model only consists of three layers, including the sapphire substrate, the n-type GaN (n-GaN), and the p-type GaN (p-GaN). The thicknesses of these three layers are *h*_sapphire_ = 100 *μ*m, *h*_n-GaN_ = 6 *μ*m, and *h*_p-GaN_ = 0.3 *μ*m, respectively. The cone array is in the form of triangle-lattice, and the major radius, minor radius, height, and periodicity are 1.5 *μ*m, 1 *μ*m, 0.5 *μ*m, and 6 *μ*m, respectively. The multiple quantum well (MQW) layer is simplified as the interface between the two GaN layers, and the effect of the backside reflector is taken into account by applying their reflectance spectra as the surface property of the backside of sapphire substrate. The reflectance spectra can be calculated using multiple thin-film theory [12], and the thickness of each layer in DBR is set to be quarter-wavelength. The power monitor is placed at 1 *μ*m distance from the top surface of p-GaN layer, and the light-extraction efficiency (LEE) is estimated by LEE = *P*_monitor_/*P*_MQW_, where *P*_monitor_ is the power detected by the monitor and *P*_MQW_ is the total power emitted from the MQW. The material parameters used in our simulation are shown in Table 1, including the materials for GaN-based LED chips (i.e., p-GaN, n-GaN, and sapphire), those for cone array (i.e., SiO_2_, polystyrene, sapphire, ZnO, and Ti_3_O_5_), and those for reflectors (i.e., SiO_2_, Ti_3_O_5_, aluminium (Al), and Ag). The LED chips with various materials of cone array and different types of reflectors are studied to optimize the LEE.

The 3D models are simulated using Monte Carlo ray tracing method [13, 14]. The ray tracing method is generally used in the case of *λ*_*m*_ ≫ *l*_*s*_, where *λ*_*m*_ is the wavelength in the material and *l*_*s*_ is the shortest optical-length in the structure. However, it can be also used to predict the trends in the case of *λ*_*m*_ that is in the same order of *l*_*s*_, since the results are similar to those estimated by 3D finite-difference time-domain (FDTD) method for nanostructures [13]. In our simulation, light rays are randomly generated from MQW at the wavelength of 460 nm and the angular distribution is Lambertian. The total number of light rays is 10 000 000, and the light rays would annihilate if their energy attenuates to be less than 5% of the initial values.

### 2.2. Experiment

The fabricated LED chips are sketched in Figure 1, and the fabrication processes are described as follows. Firstly, the epilayer was selectively etched by inductively coupled plasma (ICP) to expose the n-GaN layer. Secondly, a SiO_2_ layer was deposited by plasma-enhanced chemical vapor deposition (PECVD) as electrical barrier layer. Then, the indium-tin-oxide (ITO) transparent conductive layer was E-beam evaporated, wet-etched, and thermally annealed. Thereafter, the metal lift-off technology was used to fabricate the p-electrode and n-electrode. After that, a SiO_2_ layer was deposited by PECVD for passivation and was wet-etched to expose the p-electrode and n-electrode. Finally, the sapphire substrate was lapped and polished, and then the backside reflector would be fabricated on the bottom of the substrate.

**Figure 3:**
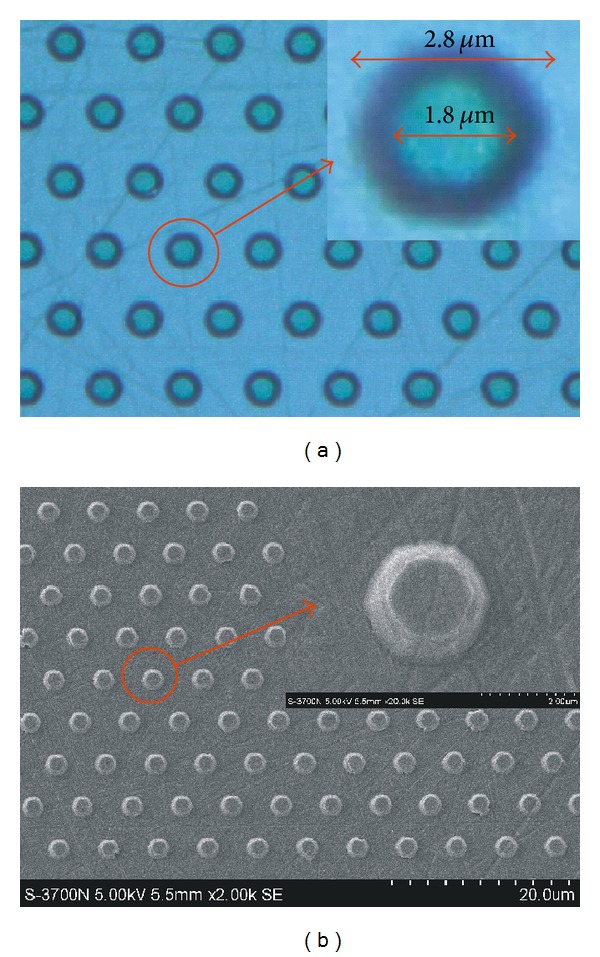
Microscope photography of fabricated SiO_2_ array taken by (a) 3D optical profiler and (b) SEM.

The 3D backside reflector consists of a triangle-lattice of SiO_2_ cone array and a Ti_3_O_5_/SiO_2_ DBR. After the sapphire substrate is lapped down to about 200 *μ*m, a SiO_2_ layer with the thickness of 500 nm is deposited by PECVD on the polished substrate. Then, the top side of the epilayer is protected by spin-coating a photoresist layer and hard-baking for 30 min at the temperature of 140°C. After that, the SiO_2_ array on the bottom of the substrate can be prepared by standard photolithography and wet-etching. The triangle-lattice in the photolithography mask is composed of hollow circles with a diameter of 3 *μ*m and a periodicity of 6 *μ*m. These parameters of triangle-lattice are limited by the resolution of standard ultraviolet lithography due to the Fresnel diffraction effect [15]. After 5 min wet-etching using buffered oxide etchant (BOE), the SiO_2_ cone array can be obtained. The surface shown in Figure 3 taken by 3D optical profiler or scanning electron microscope (SEM) is the reflecting surface, since the 16-pair Ti_3_O_5_/SiO_2_ DBR would be deposited later to cover this SiO_2_ pattern. The diameters of the bottom surface and the top surface of SiO_2_ cone are about 2.8 *μ*m and 1.8 *μ*m, respectively. The difference between the diameters of fabricated microstructures and the nominal values in the photolithography mask can be attributed to the overetching. The nonvertical sidewall with downward slope is the natural formation of long time wet-etching, since the upper part of the sidewall gets more reaction time with the etchant. Finally, the 16-pair Ti_3_O_5_/SiO_2_ DBR was deposited by ion-assisted E-beam evaporation on the backside of the SiO_2_ pattern.

In the measurements, the emission pictures observed by microscope can be used to develop the intuition of the effects of 3D reflector compared to flat reflector. The detailed optical and electrical performance are measured through probe station and integrating sphere.

## 3. Results and Discussion

### 3.1. Simulation

**Figure 4:**
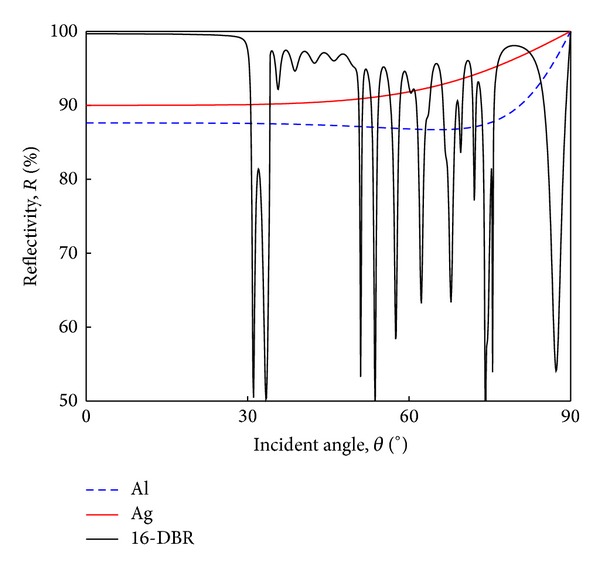
The angular reflectivity of various flat reflectors. The incident angle is measured from normal to substrate, and the reflectance spectra are calculated at the wavelength of 460 nm.

Figure 4 shows the angular reflectivity of flat Al mirror, flat Ag mirror, and flat 16-pair Ti_3_O_5_/SiO_2_ DBR (16-DBR) at the wavelength of 460 nm. It is shown that the 16-DBR exhibits higher reflectivity than the two metal mirrors, especially in the range from normal incidence to the incident angle of 30°. Take the case of normal incidence as an example; the reflectivity of flat Al mirror, flat Ag mirror, and flat 16-DBR is 87.6%, 90.0%, and 99.7%, respectively.

**Figure 5:**
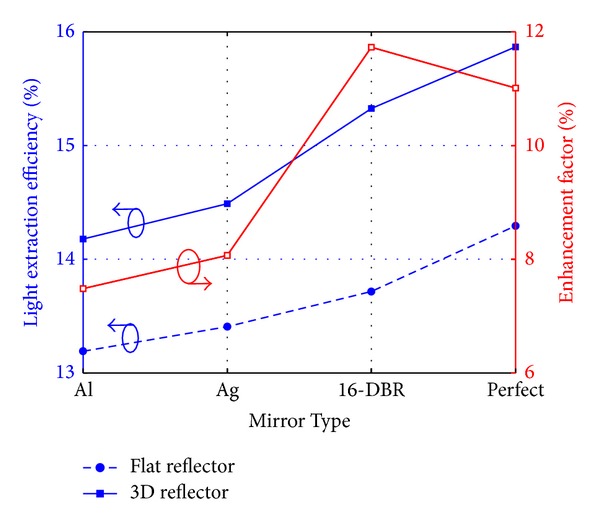
The light-extraction efficiency (left) and the enhancement factor of 3D reflectors compared to flat reflectors (right) in simulation models with different types of mirror.

The LEE of LED chips with Al mirror, Ag mirror, 16-DBR, and perfect reflector is shown in Figure 5. A perfect reflector is an imaginary mirror with the reflectivity of 100% at any wavelength and any angle. The LEE of LED chips with Al mirror, Ag mirror, 16-DBR, and perfect reflector of the flat type is 13.19%, 13.41%, 13.72%, and 14.29%, respectively, and that of the 3D type is 14.18%, 14.49%, 15.32%, and 15.87%, respectively. It is shown that the LEE increases as the reflectivity of backside reflector increases, and it can be significantly improved by 3D reflectors compared to flat counterparts. The enhancement factor, which is defined as the ratio of LEE for LED chips with 3D reflectors and that with flat counterparts, is also shown in Figure 5. The enhancement factors for Al mirror, Ag mirror, 16-DBR, and perfect mirror are 7.48%, 8.07%, 11.73%, and 11.01%, respectively. The 3D DBR exhibits the highest LEE and the highest enhancement factor compared to the two metal mirrors.

**Figure 6:**
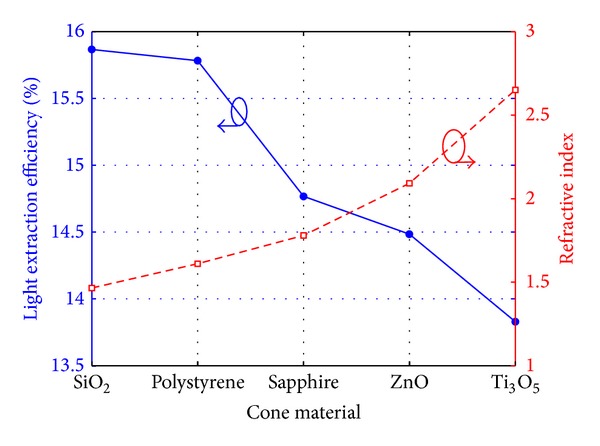
The light-extraction efficiency (left) and the refractive index (right) for different materials of cone array in simulation model.

The LEE of LED chips with 3D perfect mirror patterned by cone array with different material is also studied and shown in Figure 6. The refractive indices are tabulate in Table 1 and also shown in Figure 6. In the simulation, the extinction coefficient of Ti_3_O_5_ used for the materials of cone array is set to be 0. This is for the sake of comparison since other materials for cone array have no loss. It can be observed that the LEE decreases as the refractive index of the cone material increases. The 3D DBRs patterned by SiO_2_ cone array exhibit the highest LEE compared to those patterned by other materials.

### 3.2. Experiment

**Figure 7:**
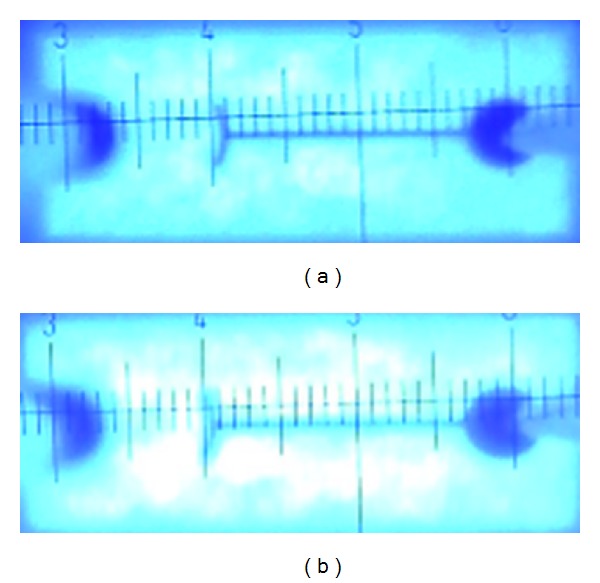
Emission pictures of LED chips with (a) flat DBR and (b) 3D DBR.

Figure 7 shows the emission pictures of LED chips with flat DBR and 3D DBR patterned by SiO_2_ cone array under the injection current of 0.1 mA. It is clearly shown that the LED chip with 3D DBR is brighter than that with flat DBR.

**Figure 8:**
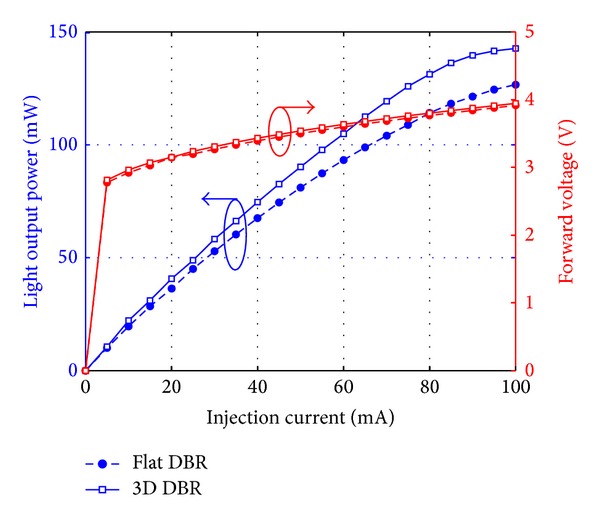
The light-output power (left) and forward voltage (right) of fabricated samples under different injection currents.

**Figure 9:**
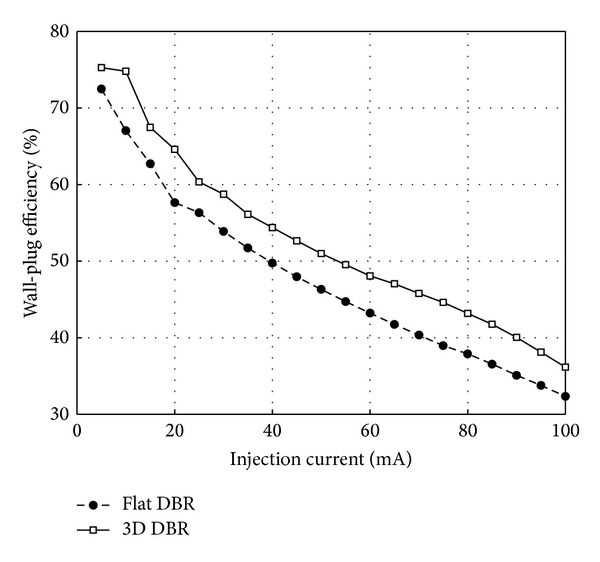
The wall-plug efficiency of fabricated samples under different injection currents.

The light-output power and the forward voltage at different injection current are shown in Figure 8. Under the injection current of 20 mA, the light-output power and the forward voltage of the LED chip with flat DBR are 36.27 mW and 3.146 V, while those with our proposed 3D reflector are 40.69 mW and 3.150 V, respectively. It is shown that the light-output power is enhanced by 12.2%, which is attributed to the larger probability of finding the escape cone for the light reflected by the 3D DBR compared to the flat DBR. On the other hand, the forward voltage has almost no shift. This indicates that the fabrication process of the 3D reflector has little effects on the electrical performance. Thus, the 3D DBR can also provide 12.1% enhancement of wall-plug efficiency, which is shown in Figure 9. The enhancement factor of wall-plug efficiency from experiments is slightly larger than that of LEE from simulation. The reason can be attributed to the improvement of internal quantum efficiency because of reduced heat generation, the single-wavelength simulation at 460 nm, and the error in measurements.

## 4. Conclusion

In summary, the LED chips with 3D reflector patterned by a triangle-lattice of cone array were simulated using Monte Carlo ray tracing method. Several types of mirrors and various materials for cone array were studied to optimize the LEE. Simulation results show that the 3D 16-DBR patterned by SiO_2_ cone array benefits large enhancement of LEE, and thus the 3D 16-DBR was fabricated and tested. Measurement results show that the 3D 16-DBR can provide 12.1% enhancement of wall-plug efficiency, which is consistent with the simulated value of 11.73% enhancement of LEE. The geometry parameters, such as shape, size, and periodicity, of SiO_2_ cone array can be tuned to optimize the LEE further.

## Figures and Tables

**Table 1:** Material parameters used in the simulation.

	Refractive index	Extinction coefficient
p-GaN	2.45	3.66 × 10^−4^
n-GaN	2.42	3.66 × 10^−4^
Sapphire	1.78	0
SiO_2_	1.4648	0
Polystyrene	1.6106	0
ZnO	2.0919	0
Ti_3_O_5_*	2.65144	0
Ti_3_O_5_^#^	2.65144	1.4378 × 10^−3^
Al	0.644	5.58
Ag	0.144	2.56

*Parameters of Ti_3_O_5_ used for materials of cone array.

^#^Parameters of Ti_3_O_5_ used for materials of Ti_3_O_5_/SiO_2_ DBR.
